# Meta-Analysis of Dyslipidemia Management for the Prevention of Ischemic Stroke Recurrence in China

**DOI:** 10.3389/fneur.2020.483570

**Published:** 2020-11-19

**Authors:** Kang-Ning Chen, Li He, Lian-Mei Zhong, Yu-Qin Ran, Yan Liu

**Affiliations:** ^1^Department of Neurology, The First Hospital Affiliated to Army Medical University (Southwest Hospital), Chongqing, China; ^2^Department of Neurology, West China Hospital, Sichuan University, Chengdu, China; ^3^Department of Neurology, First Affiliated Hospital of Kunming Medical University, Kunming, China; ^4^Medical Affairs, MSD (China) Holding Co., Ltd., Shanghai, China

**Keywords:** ischemic stroke, dyslipidemia, risk factor, stroke recurrence, odds ratio

## Abstract

**Background:** The benefit of blood cholesterol reduction for secondary prevention of ischemic stroke remains undetermined in Chinese patients. The purpose of this meta-analysis was to determine whether lipid-lowering agents including statins, fibrates, nicotinic acid, and ezetimibe reduced the risk of recurrent stroke in ischemic stroke patients in China and whether such findings could inform treatment decisions for blood lipid-lowering treatment in China.

**Methods:** The English electronic databases PubMed, EMBASE, Cochrane Library and Chinese databases CNKI, Sino-Med, Wan Fang, and VIP were searched for studies published between January 1990 and April 2020. This meta-analysis included published data from trials that randomly assigned patients to groups treated with either blood lipid-lowering regimens or placebo. Effect comparisons were made using fixed effects model in meta-analysis and linear and spline regression were performed to identify the relative risk of stroke recurrence. The primary outcome was the reduction of total ischemic stroke events, and relative risk values were obtained using a risk prediction equation developed from the control groups of the included trials.

**Results:** Five studies including 4,999 individuals with available data met the inclusion criteria. Relative to the control groups, the pooled estimated odds ratio (OR) for recurrent stroke among those who received lipid-lowering therapy was 0.79 (95% confidence interval [CI]: 0.63–1.00). A 50% or greater reduction in low-density lipoprotein cholesterol (LDL-C) significantly reduced the risk of ischemic stroke recurrence (OR: 0.15 [95% CI: 0.11–0.20]). The overall beneficial effect of statin therapy was confirmed to prevent ischemic stroke with an OR of 0.51 (95% CI: 0.36–0.72).

**Conclusions:** Effective lipid-lowering therapy could decrease the blood LDL-C level, which had a protective effect against stroke recurrence. These results support the use of predicted baseline cerebrovascular disease risk equations to inform decisions regarding blood lipid-lowering treatment in ischemic stroke patients in China.

## Introduction

Ischemic stroke is the most common neurological disease in the elderly population (1). In China, ischemic stroke constitutes 70–80% of all incident and prevalent strokes, and the annual recurrence rate of ischemic stroke in the Chinese population is as high as 17.7% (2). In recent years, many studies have shown that dyslipidemia is an independent risk factor for ischemic stroke (3–5). Therefore, blood cholesterol regulation has become a new strategy for the prevention of ischemic stroke (4, 5).

Dysfunctional blood lipid metabolism typically involves increased levels of total cholesterol (TC), triglycerides (TG), and low-density lipoprotein cholesterol (LDL) and may also involve decreased high-density lipoprotein cholesterol (HDL) levels (6). The 5-year Stroke Prevention by Aggressive Reduction in Cholesterol Levels (SPARCL) study confirmed the role of targeting blood lipids in preventing stroke recurrence and provided substantial evidence for the use of lipid-lowering therapy in the treatment of ischemic stroke (3). Furthermore, studies have shown that elevated LDL and reduced HDL levels increase the risk of stroke (7). The Treat Stroke to Target (TST) trial evaluated the benefit of dyslipidemia management and showed that targeting a LDL cholesterol level <70 mg/dL after a recent ischemic stroke or TIA could significantly decrease the occurrence of cardiovascular events, cerebral infarction, and recurrent cerebral infarction or hemorrhage as compared with targeting a LDL cholesterol of 100 ± 10 mg/dL (8). However, consistent conclusions have not been reached regarding pharmacological interventions that raise HDL-C concentrations (7). Therefore, lowering LDL cholesterol (LDL-C) has been the major target in cardiovascular protection strategies in past decades. Moreover, despite the independent relevance of LDL-C to cardiovascular events, the relationship between serum LDL-C level and the occurrence of ischemic stroke remains controversial (9). A large prospective cohort study conducted recently revealed a significant linear relationship between serum cholesterol levels and ischemic stroke (10). However, other studies have reported that cholesterol levels are not related to stroke (11). Importantly, it remains unclear whether outcomes differ according to the intensity of lipid-lowering therapy in patients with different LDL-C levels. Therefore, differences in managing dyslipidemia, including guidelines from the European Society of Cardiology/European Atherosclerosis Society (ESC/EAS), have caused many debates and much confusion in clinical practice (12). Several studies have suggested that Asians have a higher risk of stroke recurrence and rehospitalization than non-Asians (13). Thus, the development of effective guidelines for lipid-lowering therapy is important for the Asian population.

Therefore, here we review the relationship between blood lipid levels, lipid-lowering therapies, and ischemic stroke recurrence in Chinese patients in order to provide insight for the effective use of lipid-lowering therapy for secondary stroke prevention.

## Methods

### Search Strategy

The PubMed, EMBASE, and Cochrane Library were searched along with the Chinese electronic databases CNKI, Sino-Med, Wan Fang, and VIP for studies published between January 1990 and April 2020. The language was restricted to English and Chinese. The search terms were “hypercholesterolemia,” “dyslipidemia,” “cholesterol,” “low-density lipoprotein,” and “stroke.”

### Inclusion Criteria

The PICO (Population, Intervention, Comparison, Outcome) model was used to define the clinical question for this systematic review and identify appropriate studies with clinical evidence in the literature (14). The following inclusion criteria were applied: (1) the study population consisted of Chinese patients and the study was conducted in China; (2) participants had been diagnosed with ischemic stroke with or without known risk factors (hypertension, myocardial ischemia, diabetes mellitus, and hypercholesterolemia); (3) the study was a randomized controlled trial or double-blind retrospective or prospective study; (4) lipid-lowering agents such as statins, fibrates, nicotinic acid, and ezetimibe were administered for secondary prevention of stroke with the proper dosages; (5) clinical characteristics including stroke type, mortality, and hemorrhage events were reported; and (6) the incidences of stroke, mortality and adverse events among study participants were specified or could be calculated. Publications with limited data, poor data authenticity, or missing data as well as systematic reviews, meta-analyses, non-randomized studies, and evidence-based guidelines were excluded.

### Study Screening, Data Extraction, and Quality Assessment

Two reviewers screened the articles according to the inclusion and exclusion criteria independently. A third reviewer verified the data, and any inconsistency was resolved by discussion. The recorded data included characteristics of the study and the participants, details of the lipid-lowering regimens used, lipid levels before and after treatment, outcome events, and differences in outcome data between the intervention and control groups. This meta-analysis was performed according to the Preferred Reporting Instructions for Systematic Reviews and Meta-Analyses (PRISMA) guidelines. The quality assessment for studies of diagnostic accuracy (QUADAS) was applied to determine the methodological quality of the included studies (15, 16).

### Data Analysis

Stata 14.0 and Review Manager 5.3 were employed for all data analyses. The significance level was set at α = 0.05. Heterogeneity across studies was evaluated according to the Cochran-*Q* value and *I*^2^ test. Pooled odds ratios (ORs) were calculated as an indicator of the weighted pooled risk under the fixed effects model. Briefly, the OR reflects the number of participants in a group who achieved a stated end-point divided by the number of patients who did not. Linear and spline regression were performed to determine the association between the LDL-C level and the relative risk of stroke recurrence. To mitigate heterogeneity, random effects models were adopted instead of fixed effects models.

A funnel plot was constructed *via* Begger's test to assess publication bias among the included studies, with asymmetry of the plot considered indicative of publication bias. This method is based on scatter plots of the treatment effect estimated by individual studies vs. a measure of study size or precision. In this graphical representation, larger and more precise studies are plotted at the top, near the combined effect size, while smaller and less precise studies show a wider distribution below (17). All statistical tests were two sided, and differences were considered significant if *p* < 0.05.

## Results

**Figure 1:**
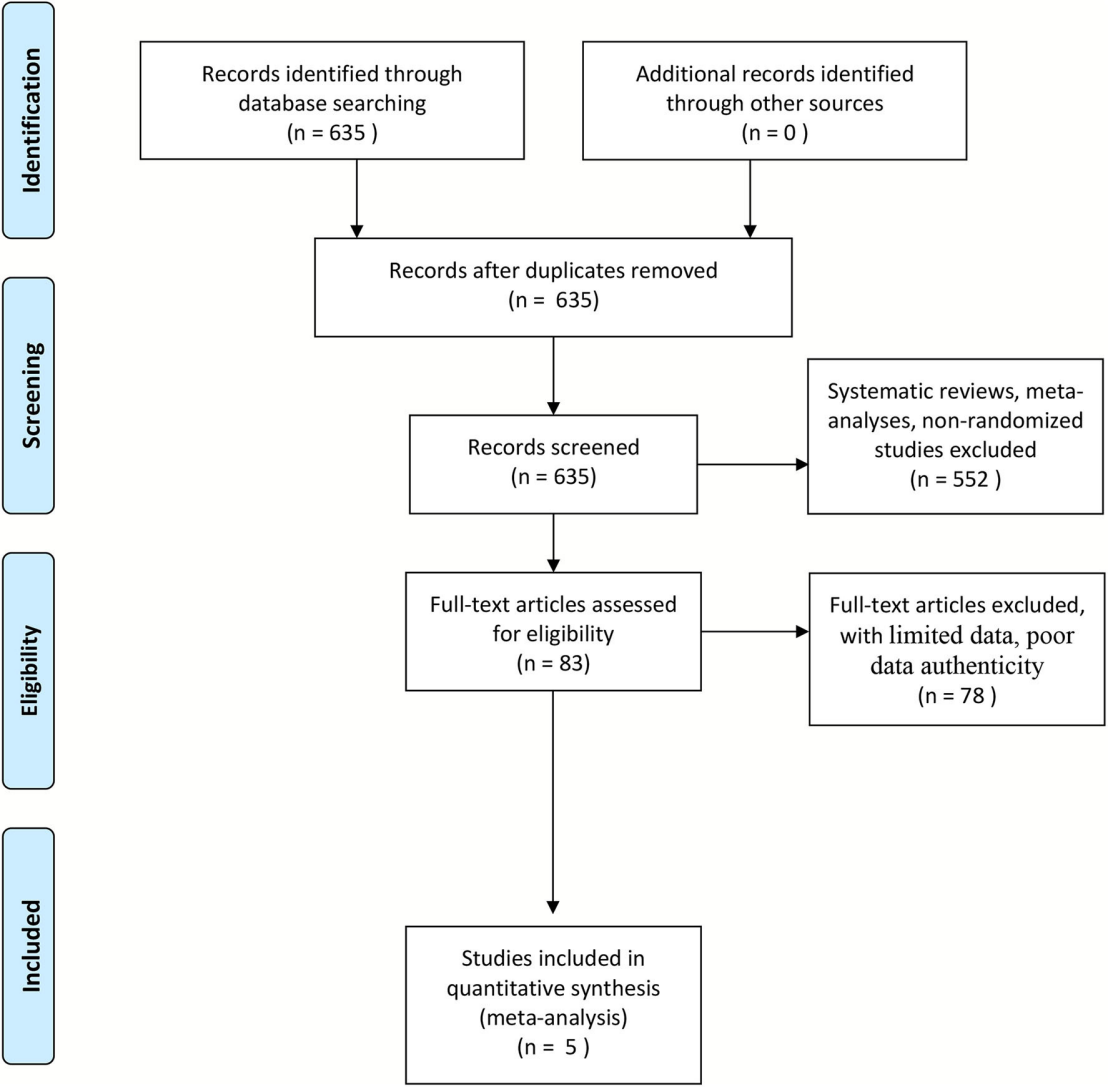
Flowchart of trial identification for meta-analysis.

In accordance with the selection criteria, we ultimately included five studies (Figure 1) (18–22). A total of 1,821 cases and 3,178 controls were included in the five cohort studies. The basic characteristics of the patient populations in the included studies are shown in Table 1. All studies were controlled for gender, age, body mass index, smoking, and other confounding factors and had adequate follow-up time to observe the occurrence of stroke (Tables 1, 2).

**Table 1:** Characteristics of included trials.

**Study**	**Sample size (F)**			**Age, years (M ± SD)**	**Stroke**			**Baseline lipid level (mmol/L)**			
	**Case**	**Control**	**Case**	**Control**	**Total**	**Case**	**Control**	**LDL**	**TC**	**TG**	**HDL**
Yan and Zhang (20)	117(45)	115(47)	58.7 ± 10.1	57.4 ± 12.4	232	7	13	4.37	6.25	3.17	1.13
Lin et al. (18)	100(26)	100(29)	61.7 ± 9.6	62.7 ± 9.8	200	17	22	4.78	2.48	6.12	0.88
Zhao et al. (21)	95(na)	95(na)	na	na	190	5	24	2.03	na	na	na
Wang et al. (19)	1,062(286)	2,806(974)	na	na	3,868	73	183	2.79	na	na	na
Zhao et al. (22)	64(39)	446(280)	60.9 ± 13.9	58.8 ± 13.7	510	53	13	3	5.07	na	na

*F, female; na, not available*.

**Table 2:** Characteristics of stroke recurrence in included trials.

**Study**	**Stroke type**, ***n***		**Treatment**		**Region of China**	**Comorbidity**
	**Ischemic**	**Hemorrhagic**	**Agent**	**Dosage (Mean, mg/d)**		
Yan and Zhang (20)	20	ns	Atorvastatin	20	Northern	Diabetes, hypertension
Lin et al. (18)	100	ns	Fluvastatin	40	Southern	Diabetes, hypertension
Zhao et al. (21)	143	47	Statins	na	Northern	Hypertension, dyslipidemia
Wang et al. (19)	1,062	ns	Pitavastatin	2	Northern	Diabetes, hypertension, dyslipidemia, carotid artery stenosis
			Rosuvastatin	9.8		
			Atorvastatin	18.5		
			Lovastatin	20.5		
			Pravastatin	23.5		
Zhao et al. (22)	53	ns	statins	na	Northern	Diabetes, hypertension, coronary heart disease, atrial fibrillation

*na, not available; ns, not specified*.

**Figure 2:**
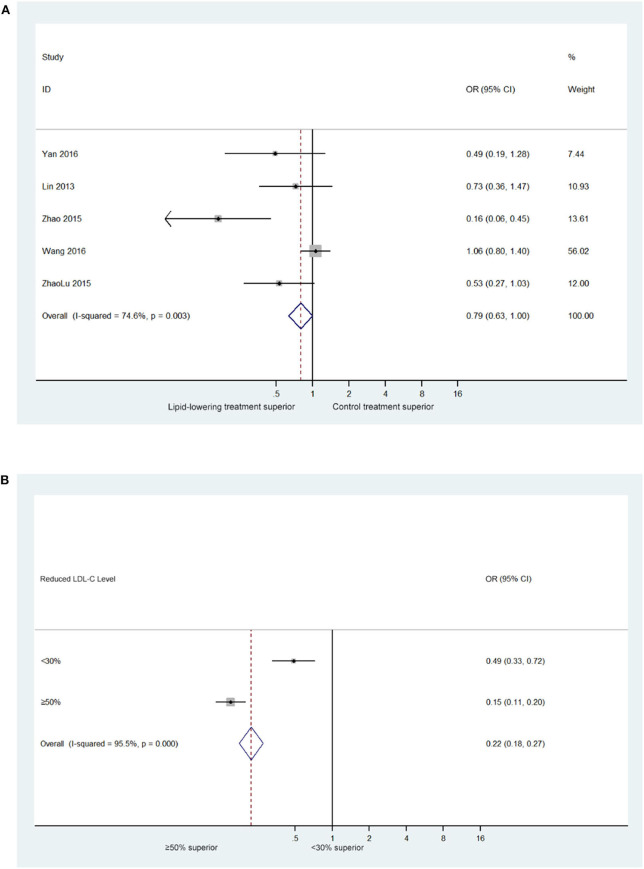
**(A)** Forest plot of estimated relative risk of stroke recurrence in trials of lipid-lowering treatment (5 trials with 1,821 ischemic stroke patients). **(B)** Standardized associations between LDL-C reduction and relative risk of stroke recurrence (5 trials with 371 ischemic stroke patients who experienced LDL-C reduction by 30–50%).

The results of meta-analysis showed that blood lipid reduction led to a significant decrease in the relative risk of recurrent ischemic stroke that were similar across all groups. There was moderate heterogeneity among the studies (*I*^2^ = 74.6%, P for heterogeneity = 0.003). On random effects analysis, the pooled relative risk with lipid-lowing treatment was 0.79 (95% confidence interval [CI]: 0.63–1.00; Figure 2A), which suggested that lipid-lowering therapies could decrease the risk of ischemic stroke recurrence.

Given that baseline blood lipid levels varied, in groups with a higher baseline level of risk, higher age may increase risk baseline of stroke recurrence. Therefore, we believe that the achieved reduction in blood lipid levels, according to the reduction ratio, could be a useful tool for comparing the effects of changes in blood lipid levels across different studies with different baseline levels. In this analysis, we found that a similar pattern of relative risk reduction across the risk groups. Moreover, our analysis showed that a >50% reduction in the LDL-C level significantly reduced the risk of ischemic stroke recurrence (0.15 [95% CI: 0.11–0.20], Figure 2B).

**Figure 3:**
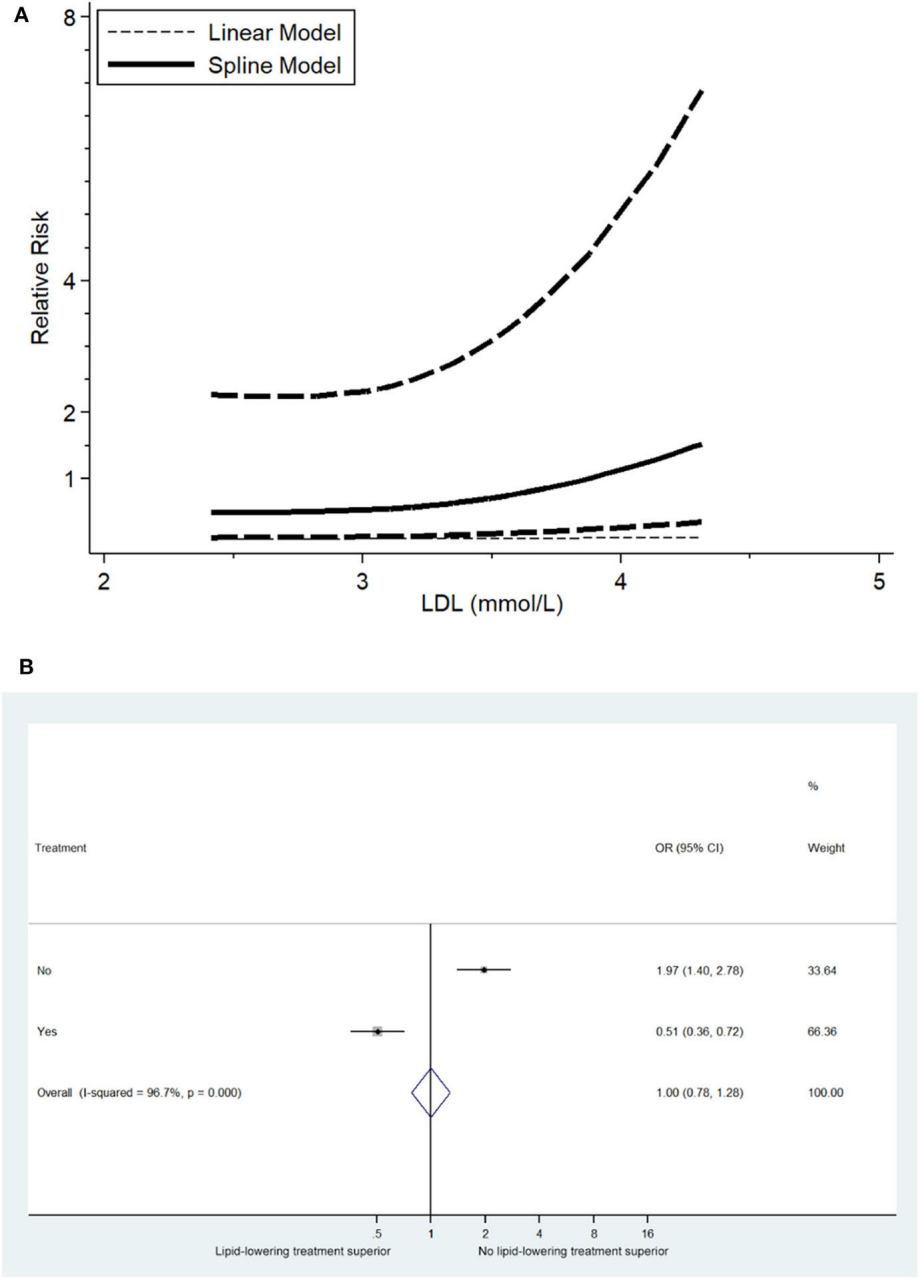
**(A)** Relevance of LDL-C level to RR of stroke recurrence (5 trials with 371 ischemic stroke patients). **(B)** Overall effects of lipid-lowering therapies on relative risk of stroke recurrence (5 trials with 1,821 ischemic stroke patients).

Furthermore, we investigated the LDL-C level-dependent effects on the risk of stroke recurrence. The results demonstrated a strong association between LDL-C level and the risk of stroke events as well as a relatively strong association between LDL-C level and the risk of ischemic stroke (Figure 3A).

To investigate the overall effects of lipid-lowering therapies on ischemic stroke recurrence, meta-analysis of the included studies was performed, and compared to no statin-treatment group of post-stroke patients, statin treatment decreased the risk of ischemic stroke occurrence (OR: 0.51 [95% CI: 0.36–0.72], Figure 3B).

**Figure 4:**
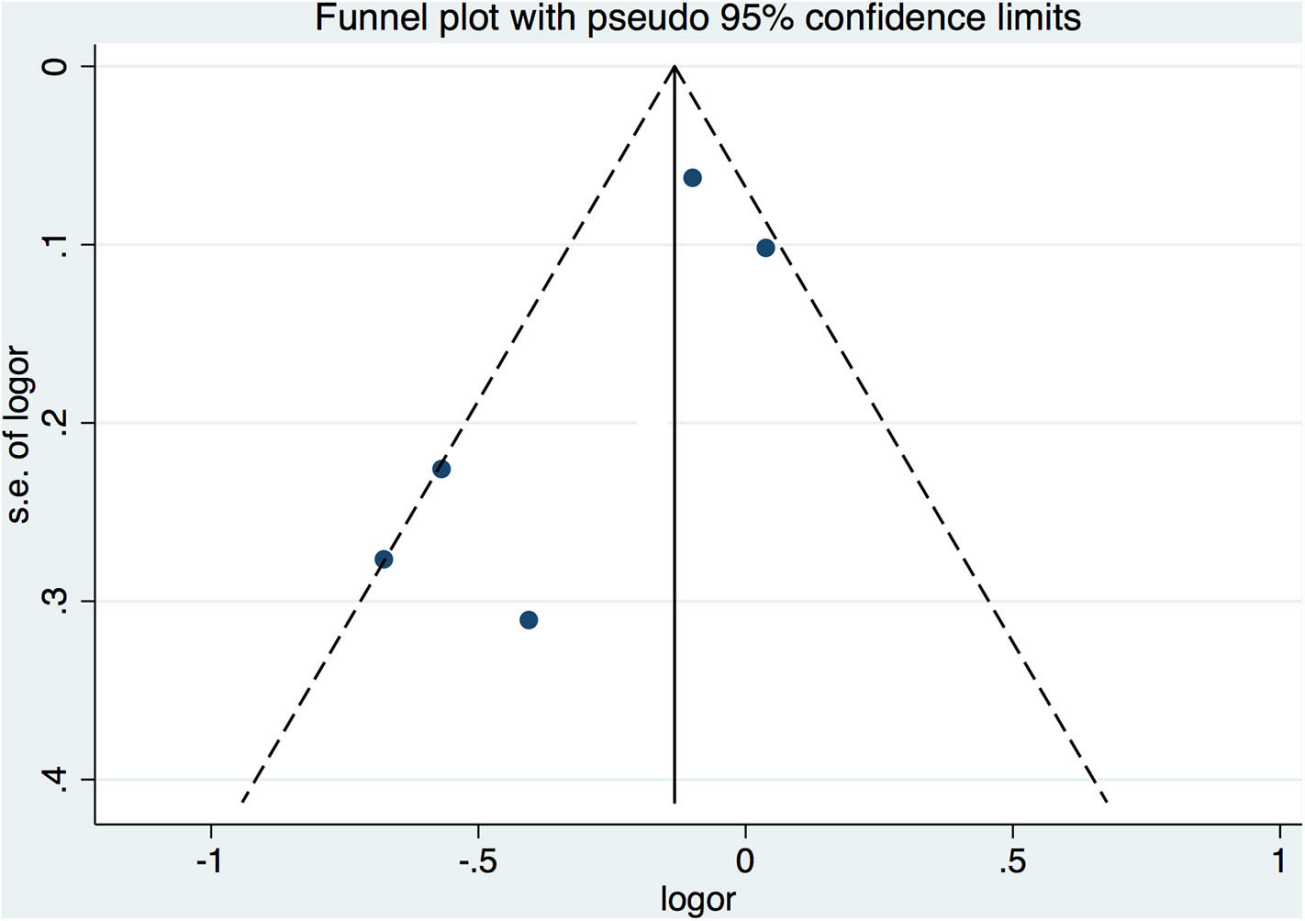
Funnel plot for the assessment of publication bias.

The funnel plot for the assessment of publication bias among the included studies is shown in Figure 4. On Begger's test, the *p*-value was 0.05.

## Discussion

Stroke is a disease derived from a variety of causes, and the major known risk factors for ischemic stroke include age, gender, smoking, obesity, hypertension, heart disease, diabetes, dyslipidemia, etc. (23, 24). Although dyslipidemia has been identified as an independent risk factor for stroke recurrence, the relationship between serum lipid levels and the occurrence of ischemic stroke remains incompletely understood, and the importance of lipid-lowering therapy in secondary stroke prevention remains to be determined.

Epidemiologically, studies also have shown that abnormal lipid metabolism is closely related to the recurrence of ischemic stroke. A single-center study in Taiwan, China that found that the risk of stroke recurrence in patients with abnormal lipid metabolism was 1.32 times higher than that in the normal control group. Another similar retrospective study in China also concluded that abnormal blood lipid metabolism is a common risk factor for both ischemic stroke and stroke recurrence.

The Stroke Prevention by Aggressive Reduction in Cholesterol Levels (SPARCL) study was the first clinical trial of statin lipid-lowering therapy for secondary prevention of stroke, and it included study population of 4,731 patients with a primary endpoint of fatal vs. non-fatal stroke and secondary end points of transient ischemic attack and other coronary events (25, 26). The results of the SPARCL study showed that lipid-lowering therapy reduced stroke recurrence in patients who achieved normal blood lipid levels (27). Recently, the TST trial demonstrated that targeting a LDL-C level <70 mg/dL (1.8 mmol/L) for 5.3 years avoided 25% of subsequent vascular events and ischemic stroke without increasing the risk of intracranial hemorrhage (8). These findings suggested that lowering LDL-C could further address the residual risks in patients with ischemic stroke.

In addition to lifestyle changes, one of the important ways to reduce the risk of ischemic stroke is to lower cholesterol levels. As one of most effective lipid-lowering medications, statins are severely inadequately used in patients with ischemic stroke in China, and the standards for drug safety need to be improved (28). The present meta-analysis confirmed that reduction of lipid levels to within normal ranges may be a critical factor for the prevention of ischemic stroke recurrence in the Chinese population. Post-stroke patients benefited from statin therapy, with these patients attaining the best possible overall outcomes. Furthermore, we identified a modest association between an elevated LDL-C level and an increased risk of ischemic stroke recurrence.

Our results indicate that a >50% reduction in the LDL-C level significantly reduced the risk of ischemic stroke recurrence. In the prevention and treatment of ischemic stroke, the intensity of lipid-regulating therapy is important, especially as statins not only reduce the LDL-C level but also atherosclerotic plaque (29, 30). Moreover, statins are widely applied for their anti-inflammatory, anti-oxidant, and neuroprotective effects (9, 31–33). However, the (34) did not recommend statin treatment targets for older adults. With regard to statin therapy for patients, the event rate for cardiovascular disease is considerably higher than that for intracerebral hemorrhage. The LDL-C/HDL-C is recognized as a strong risk predictor of cardiovascular disease, and a low LDL-C/HDL-C ratio was independently associated with an increased risk of all-cause mortality at 3 months in patients with ICH. In future studies, we should focus on the effects of lipid-regulating therapy on cardiovascular events, especially in t elderly populations (35), as well was the effects of long-term lipid-lowering therapy on the prognosis, neurological function, and survival of stroke patients (36). Notably, PCSK9 inhibitors inactivate the liver proprotein convertase subtilisin kexin 9 (PCSK9) and increase the number of LDL receptors available, leading to a profound reduction in circulating LDL particles and starting a new era of lipid-lowering drugs (37). Based on the results of the present study, we believe that serum LDL-C levels are significantly increased in patients with ischemic stroke, and a decrease in protective factors may contribute to the occurrence of stroke (26).

A consensus has yet to be reached regarding the relationship between dyslipidemia and the recurrence of ischemic stroke, and the standards for lipid abnormalities have varied among studies, limiting the ability to compare their results. In addition, most recent studies have been clinical trials or retrospective analyses, which have certain limitations. Therefore, large-scale, multi-center prospective studies are needed to further clarify the relationship between blood lipid management and ischemic stroke recurrence.

## Author Contributions

K-NC, LH, L-MZ, Y-QR, and YL conceived and designed the research. K-NC, LH, and L-MZ collected data and conducted the research. K-NC and L-MZ analyzed and interpreted data. K-NC and LH wrote the initial paper. K-NC, Y-QR, and YL revised the paper. K-NC had primary responsibility for final content. All authors read and approved the final manuscript.

## Conflict of Interest

Y-QR and YL are employees of MSD China Holding Co., Ltd. The remaining authors declare that the research was conducted in the absence of any commercial or financial relationships that could be construed as a potential conflict of interest.
