# Supplementary material for: Meta-Analysis of Dyslipidemia Management for the Prevention of Ischemic Stroke Recurrence in China
Source: Front Neurol. 2020 Nov 19;11:483570. doi: 10.3389/fneur.2020.483570 (PMC7717969; doi:10.3389/fneur.2020.483570)
Supplement: Supplementary file 1 [file Table_1.DOCX]

**Search strategy**

**S1. Literature databases.**

∙ Cochrane Central Register of Controlled Trials (CENTRAL; 2020, Issue 1)

∙ MEDLINE (Ovid SP, 1991 to 2020)

∙ EMBASE (Ovid SP, 1991 to 2020)

. CINAHL (Cumulative Index to Nursing and Allied Health Literature) (1990 to 2020)

. China National Knowledge Infrastructure (CNKI) (1991 to 2020)

. Wan Fang Data (1991 to 2020)

. Technology of Chongqing VIP database (1991 to 2020)

. SinoMed (1991 to 2020)

**S2. General search strategy**

#1 MeSH descriptor: [Hypercholesterolemia] explode all trees (25643)

#2 MeSH descriptor: [Dyslipidemias] explode all trees (78954)

#3 blood lipid (469925)

#4 MeSH descriptor: [Cholesterol] explode all trees (159033)

#5 (low density lipoprotein or high density lipoprotein):ti,ab (66079)

#6 #1 or #2 or #3 or #4 or #5 (578408)

#7 MeSH descriptor: [stroke] explode all trees (132157)

#8 MeSH descriptor: [Ischemic Attack, Transient] explode all trees (29655)

#9 MeSH descriptor: [Cerebrovascular Disorders] explode all trees (370387)

#10 #7 or #8 or #9 (375911)

#11 #6 and #10 (12293)

#12 China (1688346)

#13 (randomized controlled trial.pt. or controlled clinical trial.pt. or retrospective. or prospective. or double-blind as topic.sh. or randomly.ab. or trial.ti.) and humans.sh. (2240989)

#14 #11 and #12 and #13 (635)

#15 limit 14 to yr="1991 -Current" (635)
